# Pulmonary ct manifestations of COVID-19: changes within 2 weeks duration from presentation

**DOI:** 10.1186/s43055-020-00223-0

**Published:** 2020-06-17

**Authors:** Omar Muayad Sultan, Haider Al-Tameemi, Dhia Mahdey Alghazali, Mohammed Abed, Muthana Naser Abu Ghniem, Dhaffer Abdullah Hawiji, Enas Abdulhussain Alwateefee, Hayder Dhajir Abbas Shubbar, Amal Hussein Tauah, Nisreen Mohammed Ibraheem, Ahmed D. Abdulwahab, Raad Hefdhi Abedtwfeq

**Affiliations:** 1 Faculty of Medicine, Tikrit University, Tikrit, Iraq; 2 Faculty of Medicine, University of Kufa, Najaf, Iraq; 3 Department of Diagnostic Imaging, Al-Imam Al-Hussein Medical City, Karbala, Iraq; 4 Al-Yarmuk Teaching Hospital, Baghdad, Iraq; 5 Al-Hakeem General Hospital, Al-Najaf, Iraq; 6 AL-Hussain Teaching Hospital, Al-Muthana, Iraq; 7 Marjan City Hospital, Babylon, Iraq; 8 Al-Diwaniyah Teaching Hospital, Al-Diwaniyah, Iraq; 9 Al-Eskandaria General Hospital, Babylon, Iraq; 10 Rizgary Teaching Hospital, Erbil, Iraq; 11 Iraqi Board of Radiology and Iraqi Society of Radiology and Medical Imaging, Al-Yarmuk Teaching Hospital, Baghdad, Iraq

**Keywords:** COVID-19, Computerized tomography, Ground glass opacity, Consolidation

## Abstract

**Background:**

Coronavirus disease 2019 (COVID-19) is an infectious disease caused by severe acute respiratory syndrome coronavirus 2 (SARS-CoV-2). Chest computed tomography (CT) plays an essential role in the evaluation of COVID-19. This retrospective study aims to determine and compare the pulmonary changes in Iraqi patients with COVID-19 disease across the first two weeks after onset of symptoms using computerized tomography (CT) scan.

Ninety-six patients with COVID-19 disease were enrolled in this study. Patients were divided into two groups according to the duration of symptoms (the first group has been scanned within the first week of presentation while the second group has been scanned in the second week).

**Results:**

The CT findings in the first and second group were as follows: ground glass opacity (GGO) were 94.3% vs. 88.5%, consolidation were 25.7% vs. 34.6%, broncho vascular thickening were 18.6% vs. 7.7%, crazy paving appearance were 15.7% vs. 3.8%, tree-in-bud appearance were 4.3% vs. 10.7%, pulmonary nodules were 5.1% vs. 7.7%, and bronchiectasis were 5.5% vs. 7.7%. Pleural effusion and cavitation were seen only in the first group (2.9% and 1.4% respectively).

The distribution of CT changes across the two groups were as follows: bilateral changes were 85.7% vs. 100%; central distribution were 11.4% vs. 11.5%; peripheral distribution were 64.3% vs. 42.3%, and diffuse (central and peripheral) distribution were 24.3% vs. 46.2% while multilobar distribution were 70% vs. 80.8%.

**Conclusion:**

The type, extent, and distributions of pulmonary manifestations associated with COVID-19 infection are significantly different between the two groups who have been scanned in different stages of the disease.

## Background

In late December 2019, new pneumonia cases have emerged in Wuhan City, China, and reported to the World Health Organization (WHO). The new cases have presented with respiratory features that resembling viral pneumonia a few months before the declaration of the pandemic by WHO [1]. Those cases were caused by a virus named severe acute respiratory syndrome coronavirus 2 (SARS-CoV-2) and its disease as 2019 coronavirus disease (COVID-19). SARS-CoV-2 is a single-stranded RNA virus that belongs to order Nidovirales, family Coronaviridae, a family that also includes other viruses implied in the respiratory illnesses such as severe acute respiratory syndrome (SARS) and the Middle East respiratory syndrome (MERS) [2]. On 24 February 2020, Iraq has confirmed the first case of COVID-19 which subsequently followed by an incremental number that exceeds 1400 on 15 April 2020 [3]. The diagnosis of COVID-19 requires the detection of the specific viral genetic material in the specimens collected from nose, blood, feces or respiratory secretions, however, the variable sensitivity of this test is a problem that threatens the validity [4]. Lung injury caused by the COVID-19 infection in the form of acute respiratory distress has been seen in about 30% of cases [5]. Chest computed tomography (CT) plays an essential role in the evaluation of COVID-19 even, sometimes before the clinical symptoms become apparent [6]. Chest CT scan shows 97% and 75% sensitivity for the diagnosis of specimen positive and negative patients respectively but with only 25% specificity [7]. There is evidence of the prognostic value of chest CT which has been shown by recent studies, where a specific score by CT scan could predict the mortality of patients with COVID-19 [8]. The changes in the lung features on CT follow up have been mentioned in some previous studies [9, 10]. To have a better understanding of the chronological changes of the lung disease in COVID-19, we have performed our study to improve the accuracy of the diagnosis of the disease as no previous studies in Iraq have been performed could assess the progression of the disease on CT scan follow-up.

## Methods

### Study population

Patients’ data were collected from seven isolation Iraqi hospitals in Erbil, Baghdad, Babylon, Al-Muthanna, Najaf, and Karbala provinces, between March 10 and April 5, 2020. Ninety-six (61 males and 35 females) with proven COVID-19 infection were enrolled in this study; aged 19–82 years, with a mean age of 49.3 ± 8.3 years. All patients were symptomatic, presented with variable degrees of fever, cough, and dyspnea. The patients were divided into two groups according to the duration of symptoms, the first group (70 patients) scanned within the first week of presentation while the second group (26 patients) scanned in the second week. The COVID-19 infection was confirmed using the RT-PCR test.

### Inclusion criteria

Proved COVID-19 infection: patients with positive PCR.

Symptomatic patients presented with a variable degree of fever, cough, and dyspnea.

### Exclusion criteria

Patients with preexisting lung disease were excluded from the study.

### Study design

This study is a retrospective cross-sectional study.

### Ethics approval and consent to participate

No individual data were included in the study.

The Research Ethics Committee of the Faculty of Medicine at Tikrit University - Iraq, approved this study.

All patients included in this study gave verbal informed consent to participate in this research. If the patient was unconscious at the time of the study, their next of keen have given written informed consent.

Verbal consent is advised by our medical research ethics committee, particularly in such unusual risky situation to reduce the risk of transmission of the disease by avoiding any unnecessary contact with the positive patients.

### CT scanning protocol

A high-resolution CT (HRCT) scan was performed in all patients with 64-slice multi-detector row CT scanners (Siemens Sensation-64, Philips Brilliance-64, and GE LightSpeed-64). Patients were scanned in the supine position; head first, during breath-hold. Scanning parameters were tube voltage 100−120 kV, tube current 110−280 mA, pitch 1.375, FOV 350−400 mm. The 1.25-mm or 2.5-mm-thick images were reconstructed using a high-frequency reconstruction algorithm. All examinations were non-enhanced and no intravenous contrast medium was administered.

### HRCT image analysis

Two expert radiologists (more than 5-years experience) have evaluated the CT images separately to identify the pulmonary changes. A detailed evaluation of the images was done and pulmonary changes were identified included ground-glass opacity (GGO), consolidation, crazy paving, tree-in-bud, broncho-vascular thickening, bronchiectasis, pulmonary nodules, cavitation, and pleural effusion. The distribution of pulmonary changes was furthermore identified as unilateral vs. bilateral and central vs. peripheral distribution. Lobar distribution was reported as upper, middle/lingular, lower, and multi-lobar distribution (two or more lobes).

Lung changes were identified as peripheral if limited to the outer one-third and central when confined to the inner two-thirds of the lung. Diffuse changes when both peripheral and central zones were affected simultaneously. If there was disagreement concerning the CT analysis, a third radiologist was consulted.

### Statistical methodology

Statistical analysis was performed using SPSS 22.0. Measurement data were expressed as mean ± standard deviation, and numerical data are described as frequency. Patients were divided into two groups according to the duration of symptoms. Statistical analysis was done using ANOVA for chi-square calculation to test the significance of results regarding the CT findings across the groups, and the difference was statistically significant with a *p* value < 0.05.

## Results

### Descriptive data

Ninety-six symptomatic patients with proven COVID-19 were enrolled in this study, including 61 males and 35 females; aged 19–82 years, with a mean age of 49.3 ± 8.3 years.

Patients were divided into two groups according to the duration of the symptom as follows: the first group included the patients who scanned within the first week after onset of symptoms and the second group included the patients who scanned within the second week after onset of symptoms.

### Pulmonary CT manifestations

**Fig. 1:**
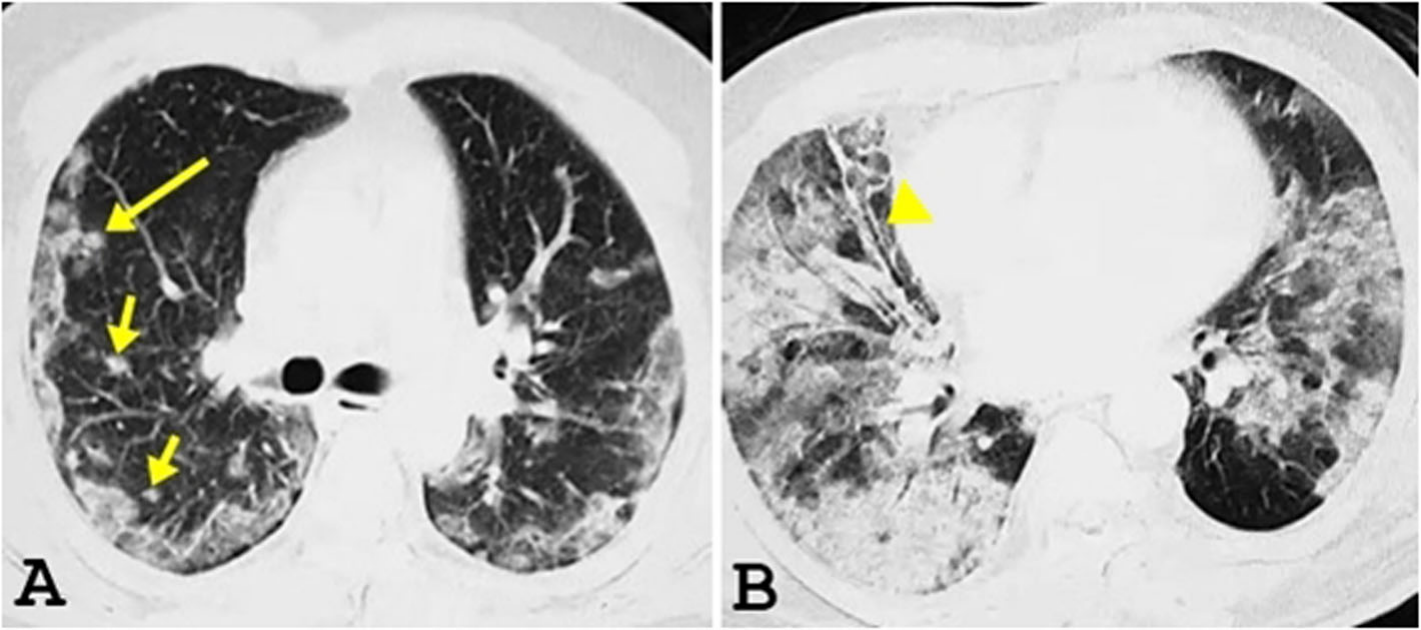
Pulmonary CT findings of COVID-19 patients. **a** An axial CT image in a 37-year-old patient in the first week, shows peripheral bilateral ground-glass opacity and few small-scattered pulmonary nodules (arrows). **b** An axial CT image in a 52-year-old patient, within the second week of presentation, shows diffuse ground-glass opacity, consolidation, and bronchiectasis (arrowhead)

**Fig. 2:**
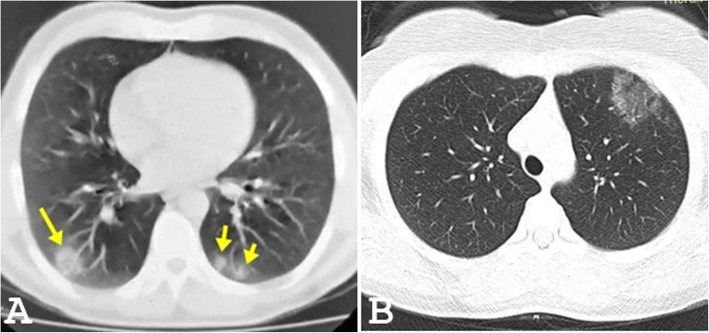
Pulmonary CT findings of COVID-19 patients. **a** An axial CT image in a 29-year-old patient shows few bilateral peripheral pulmonary nodules 3 days after cough. **b** An axial CT image in a 33-year-old patient shows single LT upper lobe peripheral ground-glass opacity, 5 days after the onset of fever

**Fig. 3:**
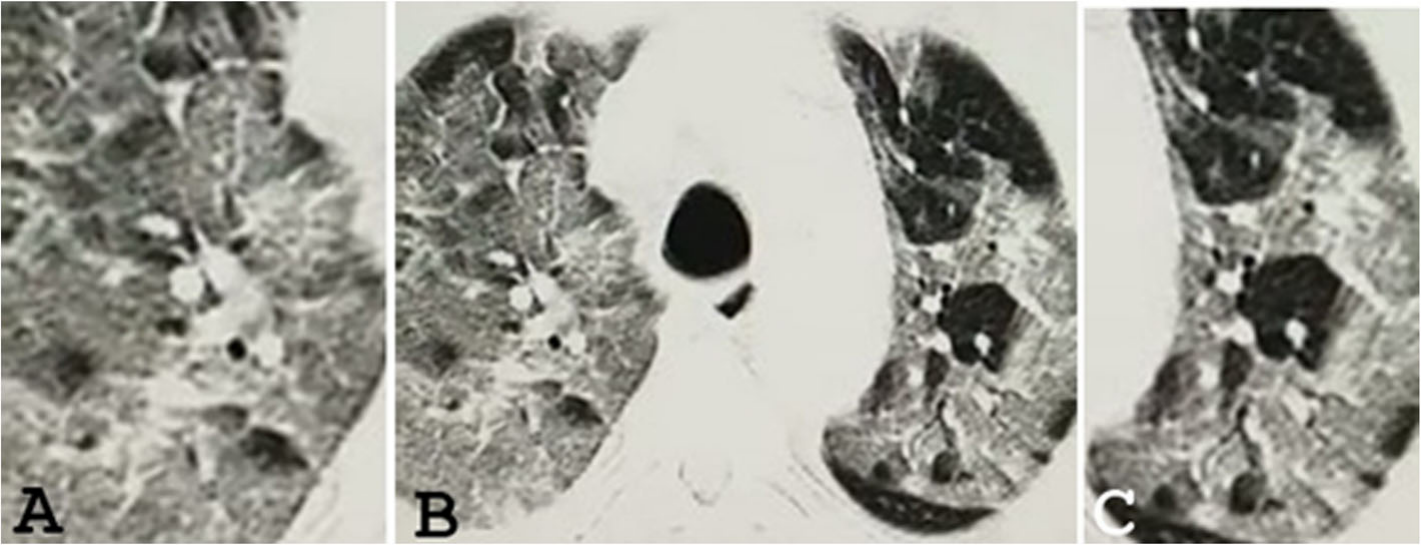
An axial CT image in a 73-year-old patient with COVID-19 infection 10 days after onset of dyspnea. **a** and **c** are magnified views highlighting the diffuse ground-glass opacity with crazy paving appearance. **b** An axial CT image shows diffuse ground-glass opacity with crazy paving appearance

GGO was noted in 73 patients (94.3%) in the first group (Figs. 1a and 2) and 23 patients (88.5%) of the second group (Fig. 1b) (significant association at *p* value = 0.05). Consolidation was noted in 18 patients (25.7%) in the first group and 9 patients (34.6%) of the second group (Fig. 1b) (little significance at *p* value = 0.05). Broncho vascular thickening was noted in 13 patients (18.6%) in the first group and 2 patients (7.7%) of the second group (little significance at *p* value = 0.05). The crazy paving appearance was noted in 11 patients (15.7%) in the first group and 1 patient (3.8%) of the second group (Fig. 3) (significant association at *p* value = 0.05). Tree-in-bud appearance was noted in 3 patients (4.3%) in the first group and 3 patients (10.7%) of the second group (not significant at *p* value = 0.05). Pulmonary nodules were noted in 4 patients (5.1%) in the first group (Figs. 1a and 2a) and 2 patients (7.7%) of the second group (not significant at *p* value = 0.05). Bronchiectasis was noted in 4 patients (5.5%) seen in the first group and 2 patients (7.7%) of the second group (Fig. 1b) (not significant at *p* value = 0.05). Pleural effusion was noted in 2 patients (2.9%) seen in the first group. Cavitation was noted in only one patient (1.4%) of the first group. Halo signs, reversed halo sign, pneumothorax, and lymphadenopathy were seen neither in the first nor in the second group (Table 1).

**Table 1:** The pulmonary CT changes of COVID-19 patients

CT characteristics	Groups		*p* value correlation
	G1 (*n* = 70)	G2 (*n* = 26)	
Ground glass opacity	66 (94.3%)	23 (88.5%)	*p* value = 0.05 significant
Consolidation	18 (25.7%)	9 (34.6%)	*p* value = 0.05 little significance
Broncho vascular thickening	13 (18.6%)	2 (7.7%)	*p* value = 0.05 little significance
Crazy paving	11 (15.7%)	1 (3.8%)	*p* value = 0.05 significant
Tree bin bud	3 (4.3%)	3 (10.7%)	*p* value = 0.05 not significant
Bronchiectasis	4 (5.5%)	2 (7.7%)	*p* value = 0.05 not significant
Nodule	3 (4.3%)	2 (7.7%)	*p* value = 0.05 not significant
Pleural effusion	2 (2.9%)	0 %	*p* value = 0.05 not significant
Cavitation	1 (1.4%)	0 %	*p* value = 0.05 not significant

### Distribution of pulmonary CT manifestations

Unilateral lung changes (Fig. 2b) were noted only in the first group (10 patients, 14.3%). Bilateral lung changes were seen in 60 patients (885.7%) of the first group (Figs. 1a and 2a) while all patients in the second group (100%) had bilateral lung changes (Figs. 1b and 3) (moderately significant at *p* value = 0.05). Predominantly, central lung changes were noted in 8 patients (11.4%) in the first group and 3 patients (11.5%) in the second group (significant association at *p* value = 0.05). Predominantly peripheral lung changes were noted in 45 patients (64.3%) in the first group (Figs. 1a and 2a) and 11 patients (42.3%) in the second group (significant association at *p* value =0.05). Diffuse (central and peripheral) lung changes were noted in 17 patients (24.3%) in the first group and 12 patients (46.2%) in the second group (Figs. 1b and 3) (significant association at *p* value = 0.05). Solitary upper lobar (Fig. 2b) changes were noted only in the first group, seen in 3 patients (4.3%). Solitary middle lobe/lingular changes were noted only in the first group, seen in 6 patients (8.6%). Lower lobar lung changes were noted in 12 patients (17.1%) in the first group and 5 patients (19.2%) in the second group (little significant association at *p* value = 0.05). Multilobar lung changes were noted in 49 patients (70%) in the first group (Figs. 1a and 2a) and 21 patients (80.8%) in the second group (Figs. 1b and 3) (strong significant association at *p* value = 0.05) (Table 2).

**Table 2:** Distribution of pulmonary CT changes among COVID-19 patients

CT characteristics (1st and 2nd week)	Groups		*p* value correlation
	G1 (*n* = 70)	G2 (*n* = 26)	
Unilateral	10 (14.3%)	0 %	*p* value = 0.05 significant
Bilateral	60 (85.7%)	26 (100%)	
Central	8 (11.4%)	3 (11.5%)	*p* value = 0.01 significant
Peripheral	45 (64.3%)	11 (42.3%)	
Diffuse	17 (24.3%)	12 (46.2%)	
Upper	3 (4.3%)	0 %	*p* value = 0.01 significant
Middle	6 (8.6%)	0 %	
Lower	12 (17.1%)	5 (19.2%)	
Multilobar	49 (70%)	21 (80.8%)	

## Discussion

With the continuing COVID-19 pandemic and the increasing number of patients suspected or confirmed with the disease, the radiologists are facing more and more cases because of the paramount role of imaging, particularly chest CT scans in the workup algorithm. Although several articles have appeared in the medical press determining variable clinical and radiological aspects of the pulmonary manifestations of the infection, there is still paucity in the studies addressing the spectrum of pulmonary changes in relation to the timing of the scan throughout the clinical course [9–11] or were just isolated case reports [12, 13]. In the current study, we compared the pulmonary radiological features associated with COVID-19 infection between two groups of patients, who underwent chest CT scans after different durations from initial clinical presentations.

In both groups, the most common observed changes were the bilateral, peripheral, and multilobar areas of GGO and lesser extent consolidation in a patchy form, which are similar to what has reported in almost all published articles yet [14, 15]. However, the proportions of changes were different between those who had CT scans within the first week (group 1) and second week (group 2), particularly the ratio of GGO and consolidation. The early CT scans within the first week showed more GGO (94.3% vs 88.5%) and lesser consolidation (25.7% vs 34.6%). This reflects the time by which pathological conversion from the interstitial edema or hyaline membrane injury early in the disease [16] to the influx of exudates and frank alveolar involvement later on in keeping with the finding of previous articles [9, 10], who also found similar changes over time after the presentation. This difference should be taken into consideration by the radiologists when interpreting suspected cases, in order not to miss the diagnosis when patients are imaged later for different causes.

Bronchiectasis, cavitation, and nodules may suggest more aggressive or superimposed infection, as well as, they already considered rare initial findings and less commonly seen or not seen in the second group possibly due to the smaller number.

The distribution of pulmonary changes was also different between the groups. The increasing bilateral pulmonary changes were seen in the second week, as all patients examined later had bilateral distribution (100% vs 85.7% in the first week) [9–11]. More axial spread (less peripheral predominance and more diffuse involvement agreed with the two recent studies [9–11] making further evidence of the current assumption that airway route is the main pathway for these viral-related pulmonary injuries. Moreover, multilobar involvement increased from 70% in early to 80.8% later in the second week, in turn signifying more pathological progression and also consistent with other studies [9, 10].

Crazy-paving pattern as results of interlobular and intralobular septal thickening superimposed over a background of GGO/consolidation, peaks at the end of the first week in one study [9] and within the second week in another study [11]. Our results are more consistent with the first one (17.9% in the first week vs 7.7% in the second week) which is possible due to the dominance of the interstitial phase. This variation in the timing of crazy-paving changes may be due to the differences in the sample size, severity of cases at presentation or the state of underlying lung parenchyma among the studies. More work with larger multi-centric and longer follow-up may resolve this issue.

It seems that no solid worldwide consensus about the exact role of CT scan in the initial diagnosis or best approach in monitoring. While some authorities have recommended the use of CT scan for initial diagnosis [7, 17], (possibly due to the shortage of RT-PCR in some centers and reduced sensitivity of this test). A multinational radiological consensus has not recommended to use imaging, including CT scan for initial diagnosis of suspected patients (unless there is a risk of progression) and to be reserved for confirmed cases with moderate-severe clinical features [18]. Therefore, CT scan is done at different times in different institutions worldwide necessitating that specific consideration to be taken for such observed difference in the appearance of CT pulmonary findings according to the time of the scans.

### Limitations

We could not determine what treatment or if any drug that may affect the findings have been given to patients during the interval between the onset of symptoms and the time of CT examination, which might be a limitation to our study. Another limitation is that changes beyond the second week might be more or less different and our study is limited to two weeks period where most of the examinations are currently performed in practice. Future studies entailing a longer duration in the recovery phase may be required.

## Conclusion

In conclusion, there is a significant difference in the pulmonary manifestations associated with COVID-19 infection when CT scan conducted earlier or later after the clinical presentation, with alteration, on one hand in the proportion and combination of GGO (becoming less) and consolidation (getting more) and, on another hand, more diffuse and multilobar distribution at the second week. These findings can help give more insight into the natural course of the disease within the first 2 weeks so that appropriate measures are taken accordingly when changes can be anticipated. Furthermore, the constellation of changes in relation to the time of scan may be of value for the radiologist to narrow the differential diagnosis and to reduce the confusion with other diseases that can mimic such appearances.

## Data Availability

The datasets used and/or analyzed during the current study are available from the corresponding author on reasonable request.

## Abbreviations

COVID-19: Coronavirus disease 2019
CT: Computerized tomography
HRCT: High-resolution computerized tomography
RT-PCR: Reverse transcriptase-polymerase chain reaction
GGO: Ground-glass opacity
